# Synthesis and Characterization of Innovative Microgels Based on Polyacrylic Acid and Microalgae Cell Wall and Their Potential as Antigen Delivery Vehicles

**DOI:** 10.3390/pharmaceutics15010133

**Published:** 2022-12-30

**Authors:** Ileana García-Silva, Miguel Olvera-Sosa, Benita Ortega-Berlanga, Víctor Ruíz-Rodríguez, Gabriela Palestino, Sergio Rosales-Mendoza

**Affiliations:** 1 Facultad de Ciencias Químicas, Universidad Autónoma de San Luis Potosí, Av. Dr. Manuel Nava 6, San Luis Potosí 78210, Mexico; 2 Sección de Biotecnología, Centro de Investigación en Ciencias de la Salud y Biomedicina, Universidad Autónoma de San Luis Potosí, Av. Sierra Leona 550, Lomas 2ª. Sección, San Luis Potosí 78210, Mexico

**Keywords:** antigen carrier, mucosal immunization, hybrid polymer, inverse emulsion

## Abstract

In this study, hybrid polyacrylic acid and *Schizochytrium* sp. microalgae (PAA/Schizo) microgels were synthesized by inverse emulsion assisted by ultrasound using the cell wall fraction as crosslinker. Physicochemical characterization of PAA/Schizo microgels revealed polymeric spherical particles (288 ± 39 nm) and were deemed stable and negatively charged. The produced microgels are not inherently toxic as cell viability was sustained above 80% when mice splenocytes were exposed to concentrations ranging 10–900 µg/mL. PAA/Schizo microgels were evaluated as antigen delivery nanovehicle by adsorbing bovine serum albumin (BSA); with a loading efficiency of 72% and loading capacity of 362 µg/mg. Overall, intranasally-immunized BALB/c mice showed null IgG or IgA responses against PAA/Schizo microgel-BSA, whereas soluble BSA induced significant humoral responses in systemic and mucosal compartments. Splenocytes proliferation assay upon BSA stimulus revealed positive CD4+ T cells-proliferation response in PAA/Schizo microgels-BSA group. Thus, PAA/Schizo microgels constitute functional antigen delivery vehicles of simple and ecofriendly synthesis. Moreover, the use of cell wall fraction as cross-linker agent provides an alternative use for the generation of high-value products using residual algae biomass from the oil industry. Our data suggests that the PAA/Schizo microgels are potential antigen delivery vehicles for immunotherapy development.

## 1. Introduction

Mucosal immunization is highly attractive as it offers easier administration compared to parenteral routes, is more comfortable for patients, and allows the induction of effective mucosal immune responses. In particular nasal vaccination is advantageous since the nose is an accessible tissue with a large absorption surface due the presence of numerous microvilli, allowing the induction of both mucosal and systemic immune responses as consequence of the function of the nose-associated lymph tissue [1]. Therefore, nasal vaccines are a practical approach for large scale immunization campaigns, avoiding the use of needles, which decreased costs and lower risks associated with vaccine administration.

Several studies have focused on nasal immunization against infectious and non-infectious diseases, such as influenza [2], hepatitis B [3], atherosclerosis [4], Alzheimer’s disease [5], among others. However, mucosal vaccination often suffers from low efficacy due to weak antigen adsorption, which is caused by the antigen dilution and degradation and the short retention time onto the nasal mucosal membranes due to mucociliary clearance, and reduced capacity to cross the mucus barrier [6]. To overcome these limitations, the use of carriers for antigen delivery has been proposed as an approach to protect the antigen from dilution or degradation and increase its residence on mucosal surfaces. The use of lipid-based systems, such as micro- and nanoemulsions, or polymeric materials, including chitosan or acrylic acid, has been assessed to enhance the efficacy of mucosalvaccines [7,8,9,10,11]. 

Emulsions are colloidal systems of two immiscible liquid phases that are classified in water-in-oil (W/O, in which the oil acts as the continuous phase where the aqueous phase is dispersed forming small droplets), and oil-in-water (O/W, in which the oil is dispersed in the aqueous phase) [12]. Based on the droplets size, emulsions can be identified as micro- or nanoemulsions, both of which have been used in the encapsulation of hydrophobic bioactive molecules to assess them as drug delivery vehicles in view to improve absorption and bioavailability [13,14,15]. In the vaccinology arena, emulsions have been investigated primarily as adjuvant and antigen delivery agents in human and animal vaccine formulations [16,17,18], however W/O formulations have resulted highly reactogenic due to high oil content. Although squalene O/W-based emulsions have been licensed in some countries, rational selection of oil composition and surfactants remains as major concern in vaccine development [19,20]. Another important aspect that limits the use of emulsions as antigen delivery systems is their short shelf-life and their low stability, specially for nanoemulsions [21,22]. To overcome these limitations, high concentrations of surfactants, which lower the surface tension, and co-surfactants are required. Alternatively, polymeric particles stand as a promising approach to improve stability and extend antigen release [23,24,25].

Polymeric microgels are a class of nanocarrier systems which are composed of nanometric networks assembled with amphiphilic or hydrophilic natural or synthetic polymers [26]. Microgels stand as promising materials for the development of effective antigen delivery vehicles [27]. These 3D-nanonetworks provide a large surface area that is available for biomolecular conjugation mediated by the functional groups exposed to their surface. Furthermore, microgels are attractive materials because they possess a tunable size, are easily prepared, have minimal toxicity and show a stimuli responsiveness and stability in the presence of serum. In addition, microgels are capable to retain a high amount of water without losing their structure and they have relatively high drug encapsulation capacity [28]. 

Microgels can be physically or chemically crosslinked, being the latter the approach that renders the most stable materials upon pH and temperature variations [29]. Among chemical crosslinkers, glutaraldehyde and formaldehyde are predominantly used. Nevertheless, these molecules could induce cytotoxicity that limits their use in biomedical applications [30]. Another issue is given by the requirement during synthesis (inverse emulsion) of organic solvents (e.g., cyclohexane, hexadecane, and hexane) to induce polymer precipitation, which implies special safety measures and residues treatment [31,32]. Therefore, the development synthesis methods based on non-toxic cross-linking agents and natural oil medium are highly desired.

Natural polymers, including chitosan and alginate, have been widely explored as vehicles for the encapsulation and delivery of bioactive molecules. Nanomaterials based on these polymers show good biocompatibility, mucoadhesive properties and pH-responsiveness in mucosal drug delivery applications [33,34]. Several synthesis methods have been reported including hydrodynamic electrospray ionization jetting which allows control in particles size [35,36,37]. Synthetic polymers, such as polyacrylic acid have been used for the development of stimuli-responsive microgels (NGsPAA); these specific 3D nanonetworks are characterized by their response to changes in external conditions by changing their physicochemical properties [38]. NGsPAA are anionic polyelectrolytes with good biocompatibility that display pH [39] or temperature-responsiveness [40] and can be applied as drug or antigen delivery vehicles [41,42,43]. 

However, in the vaccinology field there is a need to expand the portfolio of the available materials used for antigen delivery having innovative properties, such as enhanced stability, lower cost, ecofriendly synthesis, and immunostimulatory activity. Hybrid microgels based on natural and synthetic polymers have been proposed for biomedicals applications since these synergistically combine the properties of the individual components. In these emergent systems, natural polymers provide biocompatibility, low-toxicity, biodegradability, and bioactivity, while synthetic polymers improve the stability of the polymer nanostructure [44]. Among the natural polymers used for this purpose are proteins and polysaccharides obtained from different organisms, such as fungi and algae [45].

Microalgae are organisms with many biotechnological applications. The cell wall from these organisms is a source of several polysaccharides with attractive properties, such as mucoadhesive and immunostimulatory activities; some examples include k-carrageenan [46,47], alginate [48] and β-D-glucan [49,50], among others. Interestingly, some of such compounds exert adjuvant properties, which make them attractive in vaccine development [51,52]. 

*Schizochytrium* sp. is a marine microalga used at the industrial level for the production of high-value products, such as docosahexaenoic acid (DHA). However, the residual biomass obtained following oil extraction have not been used for the development of high-value products; it is currently used as feed for livestock [53]. A few efforts have been made to give this residual product a more valuable application. For instance, Yin et al. [54] proposed the use of *Schizochytrium* sp. residues as a culture media supplement during fermentation to produce DHA. *Schizochytrium* sp. biomass is a potential sustainable material thus in this study the use of *Schizochytrium* sp. cell wall fraction (CWF) as a crosslinker agent for the preparation of microgels is proposed. The proof of the concept was performed by selecting PAA as a suitable polymeric matrix. The microgels based on PAA and CWF from *Schizochytrium* sp. (PAA/Schizo) were obtained by inverse emulsion method assisted by ultrasound, rendering a new, simple and ecofriendly synthesis method, which involves the use of a natural oil (coconut) that facilitates the production of a toxic compounds-free material. Synthesis parameters including polymer concentration, amplitude percentage and sonication time were investigated in order to obtain uniform particles with low polydistribution. The performance of PAA/Schizo microgels to deliver antigens was evaluated in BALB/c test mice using bovine serum albumin (BSA) as model antigen, providing evidence on a differential immune response when this carrier was used. The in vivo evaluation suggests that PAA/Schizo microgels can be potentially applied as antigen delivery vehicles as these induced a differential immune response against BSA.

## 2. Materials and Methods

### 2.1. Cell Wall Fraction (CWF) Isolation

*Schizochytrium* sp. (strain ATCC 20888) was cultured at 25 °C in modified seawater medium prepared as follows: 1 g/L yeast extract (BD Bioscience, San Jose, CA, USA), 0.2 g/L FeSO_4_, 5 g/L peptone (MCD LAB, Edo. Mex., Mexico), 15 g/L agar (MCD LAB, Edo. Mex., Mexico) and 35 g/L NaCl (Karal, Gto., Mexico). 679BY medium, which contained 1 g/L yeast extract, 1 g/L peptone, 5 g/L dextrose, and 35 g/L NaCl, was used for liquid cultures, which were incubated at 28 °C and 150 rpm. After 8-day incubation, *Schizochytrium* sp. biomass was recovered by centrifugation at 5500 rpm for 5 min; and the supernatant was discarded. An ultrasound probe (model GEX130 PB, Sonics & Materials Inc., Newtown, CT, USA) was used for cell disruption; the biomass was sonicated for 10 min at 60% amplitude (pulses of 30 s with 30 s delay in between) followed by 4 min at 90% amplitude and then it was centrifuged at 5500 rpm for 20 min at 4 °C (Centurion Scientific, West Sussex, UK). The supernatant was discarded and the cell wall fraction (CWF) was washed twice with 5 mL deionized water. CWF was dried in an oven at 50 °C and it was subsequently pulverized in a ceramic mortar until a fine powder was obtained. Then, the CWF was stored at room temperature until further use. 

### 2.2. Synthesis of PAA/Schizo Microgels

Poly (acrylic acid)/*Schizochytrium* sp. CWF (PAA/Schizo) microgels were synthesized by the inverse emulsion (water-in-oil (W/O)) method assisted by ultrasound. Synthesis was adapted from the previously reported methodology by Mousaviasl et al. [50]. Previous to the emulsion, PAA polymers were synthesized in water-borne system by free radical polymerization using acrylic acid monomer (purity 99%, Sigma-Aldrich, St. Louis, MO, USA) and 2,2′-Azobis (2-methylpropionamidine) dihydrochloride (purity 97%, Sigma-Aldrich, MO, USA) as initiator. The total concentration of the acrylic acid monomer in the reaction solution was 25% (*w/w*). The amount of initiator was 0.5% (*w/w*) in relation to the monomer. PAA synthesis was carried out at 60 °C and vigorous stirring, and the reaction was finished when an increase in viscosity was observed. PAA was washed five times with deionized water and then dried in an oven at 50 °C. Coconut oil (purity 99%, Carco productos, San Luis Potosí, México) was used to formulate (w/o) inverse emulsion. 32.5 mL of coconut oil were mixed with 3 g of Polyglycerol polyricinoleate (PGPR) (donated by Palsgaard^®^ company, San Luis Potosí, México) to form the continuous phase. The mixture was sonicated in an ultrasonic bath for 5 min to uniformly disperse the surfactant in the oil phase. The aqueous phase solution was prepared by dissolving 12 mg of PAA in 6.6 mL of distilled water. The PAA suspension was dropwise added to the continuous phase under vigorous magnetic stirring, and 6 mg of CWF were added. The PAA/Schizo emulsion was homogenized for 2 min at a 30% amplitude using a high energy ultrasound probe (Ultrasonic processor VCX 750 Watt, Sonics vibra-cell ™, Sonics & Materials Inc., Newtown, CT, USA); in this step the sample heating was minimized by using a cool water bath. Covalent bonding between PAA and *Schizochytrium* sp. CWF was subsequently promoted by adding 62 mg of 1-ethyl-3-(3-dimethylaminopropyl) carbodiimide hydrochloride (EDC, commercial grade, Sigma-Aldrich, St. Louis, MO, USA) as carboxyl activator agent. The ultrasound treatment was continued for 20 min at a 30% amplitude. The produced microgel emulsion was precipitated by adding 50 mL of acetone dropwise under vigorous magnetic stirring. The precipitate was collected by centrifugation at 4855 RCF for 20 min. The microgels pellet was serially washed with absolute ethanol, distilled water, and 70% ethanol, with centrifugation and removal of supernatant steps in between. Microgels were stored at 4 °C until further analysis.

### 2.3. Characterization

#### 2.3.1. Transmission Electron Microscopy

The morphology and particle size of the PAA/Schizo microgels were analyzed using transmission electron microscopy (TEM) with a JEOL-2100 HRTEM equipment operated at 80 kV (JEOL Ltd., Tokio, Japan). A microgel sample was resuspended in water, placed on a copper grid, and let dry at room temperature (CF200-Cu 200 mesh, Electron Microscopy Sciences, Hatfield, PA, USA). 

#### 2.3.2. Hydrodynamic Diameter and Zeta Potential Measurement

The mean diameter and size distribution of the PAA/Schizo microgels were measured by dynamic light scattering (DLS) using a Zetasizer Nano ZS (Malvern Instruments, Malvern, UK); the average particle size (ZAve) and polydispersity index (PDI) were determined using an aqueous fresh dispersion of microgels. All measurements were performed at 25 °C in triplicate. The microgel stability was evaluated by determining the ZAve and PDI of samples over 9 h at 25 and 37 °C at different pH values. The zeta potential of the sample was measured at pH 1.2, 4.6, 6, 7.4 and 8.4 using the Zetasizer Nano ZS (Malvern Instruments, Malvern, UK) in folded capillary cells (Malvern, UK). 

#### 2.3.3. Fourier Transform Infrared Spectroscopy 

The chemical structure of PAA/Schizo microgels and the evolution of the nanomaterials in their various modification steps were followed by Fourier transform infrared spectroscopy (FT-IR) using an Agilent Cary 600 series FT-IR instrument coupled with an attenuated total reflectance (ATR) accessory (Agilent Technologies, Santa Clara, CA, USA). The spectra were recorded in the range of 400 cm^−1^ to 4000 cm^−1^ with 4 cm^−1^ resolution and 32 scans. 

#### 2.3.4. Thermogravimetric Analys

Thermogravimetric analysis of CFW, PAA and PAA/Schizo microgels was carried out in a 550 TGA (TA Instruments, New Castle, DE, USA). Samples were placed on alumina pans and thermal decomposition was performed from 30 to 450 °C with a heating range of 10 °C/min under nitrogen gas flow (60 mL/min). 

### 2.4. Preparation of BSA-Loaded PAA/Schizo Microgels

Bovine serum albumin (BSA) (purity > 98%, Equitech-Bio Inc., Kerrville, TX, USA) loading capacity of the PAA/Schizo microgels was determined by the incubation method. Concisely, 1 mg of microgels was mixed with a BSA solution at 1 mg/mL in PBS at pH 7.4 and 6, and incubated for 36 h at 4 °C under stirring. Microgels were separated from the aqueous medium containing free BSA through centrifugation at 14,000 rpm for 20 min. The amount of free BSA in the supernatant was measured at different time points by the Lowry assay using a spectrometer at 550 nm. The BSA loading efficiency (*LE*) and loading capacity (*LC*) of the microgels were calculated as follows [41]:

$$LE\;\left(\%\right)=\left(\frac{Total\;amount\;of\;BSA-Free\;BSA}{Total\;amount\;of\;BSA}\right)\times 100\;LC=\frac{Total\;amount\;of\;BSA-Free\;BSA}{Dry\;nanogel\;weight}$$

### 2.5. In Vitro Cell Viability

Cytotoxicity of the PAA/Schizo microgels was evaluated in mouse splenocytes by the resazurin assay. Mice splenocytes were obtained following the protocol reported by Govea-Alonso et al. [55], and cell viability was estimated by trypan blue staining. 1 × 10^6^ splenocytes were seeded in 24-well culture plates and maintained in RPMI (Sigma-Aldrich, St. Louis, MO, USA) medium supplemented with 1 Mm sodium pyruvate (Sigma-Aldrich, St. Louis, MO, USA), 0.1 Mm non-essential amino acids (Sigma-Aldrich, St. Louis, MO, USA), 2 Mm glutamine (Sigma-Aldrich, St. Louis, MO, USA), 25 Mm HEPES (Purity > 99.5%, Sigma-Aldrich, St. Louis, MO, USA), 100 U/Ml penicillin (IBI Scientific, Peosta, IA, USA), 100 µg/mL streptomycin (IBI Scientific, Peosta, IA, USA) and 10% (*v/v*) fetal bovine serum (FBS, Gibco BRL, Grand Island, NY, USA). PAA/Schizo microgels at different concentrations (10, 100, 500 and 900 µg/mL) were added and cultures were incubated for 24 h at 37 °C in a 5% CO_2_ atmosphere. Splenocytes without microgels were used as positive control, whereas cells treated with DMSO (Sigma-Aldrich, St. Louis, MO, USA) were used as negative control. After incubation, cells were washed and incubated with resazurin (30 µg/mL) during 72 h. Fluorescence was measured (Ex = 560 nm and Em = 590 nm) using a FlexStation II equipment (Molecular Devices, San Jose, CA, USA) and the SoftMax Pro software. Samples were analyzed in triplicate.

### 2.6. Immunization Study

Six groups (n = 4) of 8–12-week-old male BALB/c mice were randomly established and subjected to intranasal (i.n.) administration with one of the following treatments: 50 µg of soluble BSA, 50 µg of BSA embedded in PAA/Schizo microgels, 10 µg of soluble BSA, 10 µg of BSA embedded in PAA/Schizo microgels, 10 µg of BSA plus 1 µg of Cholera toxin (CT, Sigma-Aldrich, St. Louis, MO, USA) as adjuvant, or PAA/Schizo microgels in PBS. PBS was used as the vehicle (pH = 7.4). Mice were nasally immunized on days 1, 14 and 39 and blood samples were collected on days −1, 13, 21, and 53. Sera were separated by centrifugation at 5000 rpm for 10 min and stored at −20 °C until further use. Mice were sacrificed by cervical dislocation 2 weeks after the last immunization. Spleens were collected and used for proliferation assays described in the previous section. For nasal washes, the jaw of euthanized mice was carefully removed until the trachea showed up, blood present was cleaned using cold PBS. Afterwards, a 19 G needle was inserted into the trachea and the mice were positioned vertically to a 1.5 mL collection tube. 200 µL of PBS containing 1 mM phenylmethylsulfonyl fluoride (PMSF, Sigma-Aldrich, St. Louis, MO, USA) was flushed through the needle, passed the mice nasal cavity and collected from nostrils into the tubes. During the collection it was ensured that PBS did not escape through the oral cavity of the mice by keeping the needle in the direction of the back of the trachea avoiding making a second hole. The fluid recovered from the nostrils was used for antibody content analysis right after collection [56,57]. This protocol was approved by the Committee on Research Ethics from the Faculty of Chemistry/University of San Luis Potosi (Permit Number: CEID-2015069).

### 2.7. Enzyme-Linked Immunosorbent Assay (ELISA)

ELISA was performed to determine the presence of anti-BSA IgG antibodies in serum and anti-BSA IgA antibodies in nasal washes. 96-well polystyrene plates were coated overnight at 4°C with BSA (1 µg/well) in carbonate buffer (15 mM Na_2_CO_3_, 35 mM NaHCO_3_). Then, plates were washed three times with PBS-Tween (PBS-T) and blocked with 5% fat-free dry milk dissolved in PBS at room temperature for 2 h. After washing, diluted serum samples (1:20) and nasal washes (undiluted) were added and plates were incubated overnight at 4 °C. Samples were analyzed in triplicate. Plates were washed and goat horseradish peroxidase-conjugated anti-mouse IgG or IgA was added as a secondary antibody (1:2000). After incubation for 2 h at room temperature, plates were washed and a substrate solution composed of 0.3 mg/mL 2,2′-azino-bis(3-ethylbenzothiazoline-6-sulfonic acid) (ABTS, Sigma-Aldrich, MO, USA) and 0.1 mM H_2_O_2_ was added. After 30 min incubation, optical density (OD) values at 405 nm were measured using a Thermo Scientific Multiskan^®^ FC microplate photometer (Thermo Scientific, USA).

### 2.8. Flow Cytometry

Flow cytometry was performed to measure the percentage of CD4^+^ T cells in splenocytes from immunized mice. Splenocytes were obtained as described above and cultured in 24-well plates, with a 1 × 10^6^ splenocytes/mL initial density in RPMI medium. Cells were stimulated or not with 2.5 mg/mL BSA and incubated for 65 h at 37 °C in a 5% CO_2_ atmosphere. Cells were subsequently harvested, washed, and stained with phycoerythrin (PE)-conjugated anti-CD4 antibody (BD Biosciences, San Jose, CA, USA) for 20 min (4°C) and then fixed with 1% paraformaldehyde. Unstained cells were used to compensate the background autofluorescence. Cells were washed, resuspended in PBS, and acquired on a BD FACScanto II™ cytometer (BD Biosciences, San Jose, CA, USA). Results were analyzed in the FlowJo software.

### 2.9. Statistical Analysis

Data were presented as mean ± standard deviation (SD). Significant differences in antibody levels and cell viability values between pairs of groups were assessed using one-way analysis of variance (ANOVA) followed by mean comparisons using Tukey’s test (*p* > 0.05) in the GraphPad Prism software.

## 3. Results

### 3.1. Production of Stable PAA/Schizo Microgels

Microgels constitute attractive materials for the development of innovative vaccines, especially for those administered by mucosal routes. This study was focused on the development of a microgel for nasal immunization using algae cell wall fractions, which is a low-cost material that at the industrial level is not used for the generation of high-value products. Hence, PAA/Schizo microgeles were synthesized by inverse emulsion assisted by ultrasound using an aqueous phase containing PAA and CWF, emulsified in coconut oil phase containing PGPR as surfactant. 

**Figure 1:**
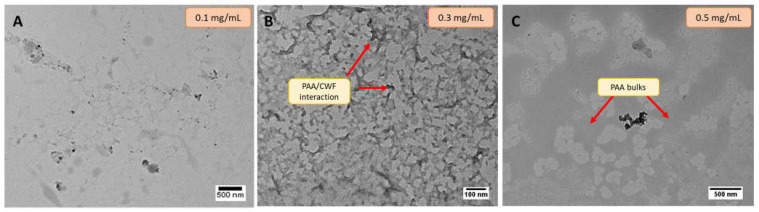
Transmission electron microscopy (TEM) micrographs of reactions carried out in aqueous medium using different concentrations of polyacrylic acid (PAA): (**A**) 0.1 mg/mL, (**B**) 0.3 mg/mL (red arrows indicate PAA bulks), (**C**) 0.5 mg/mL (red arrows indicate interaction between PAA and *Schizochytrium* sp.); maintaining constant the following parameters: *Schizochytrium* sp. cell wall fraction (CWF) (6 mg), ultrasound time (5 min), and amplitude (20%).

The impact of synthesis parameters and the viability of using ultrasound treatment to break the bulk PAA polymer synthesized by free radicals was first determined. The evaluated parameters were initial concentration of PAA polymer (0.1, 0.3, 0.5 mg/mL), ultrasound time (5 and 20 min) and amplitude (20, 40%). The goal in this step was to obtain spherical shape polymeric nanoparticles with a narrow size distribution. In the first stage, the synthesis of microgels was performed in aqueous solution to determine the impact of this innocuous medium and the influence of initial parameters in particle size and shape. Figure 1 shows TEM images of PAA/Schizo hybrid polymers obtained at various initial concentrations of PAA, keeping constant the ultrasound period and amplitude (5 min and 20%, respectively). From Figure 1A it can be noticed that at low PAA concentration (0.1 mg/mL) any structured polymer is formed; in fact, cell debris were observed separated from the PAA polymer, which is likely due to the lack of reaction between PAA polymer and microalgae cell walls under this reaction conditions. Figure 1B shows the morphology of the polymeric material obtained when PAA was used at 0.3 mg/mL, which consists of a polymeric material with more structured and condensed regions, suggesting an interaction between PAA and *Schizochytrium* sp. CWF. At high polymer concentration (0.5 mg/mL) aggregates of bulk polymer were found (Figure 1C). Based on these results the selected PAA concentration for further experiments was 0.3 mg/mL. 

**Figure 2:**
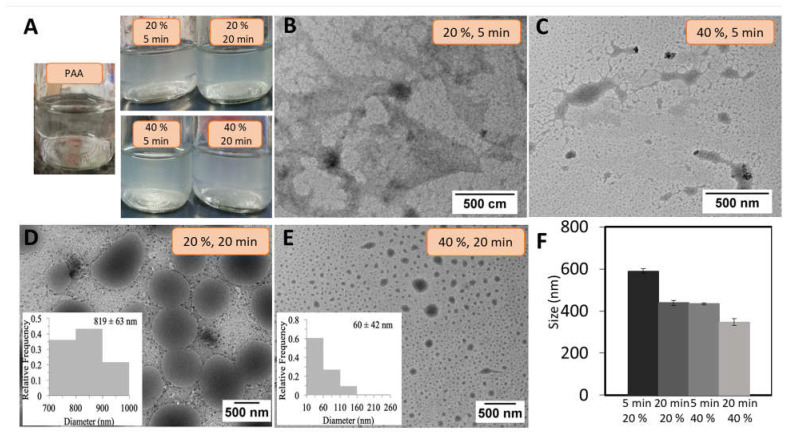
(**A**) pure polyacrylic acid (PAA) (control) and polyacrylic acid/*Schizochytrium* sp. (PAA/Schizo) microgels suspensions obtained at various synthesis conditions. Transmission electron microscopy (TEM) micrographs of PAA/Schizo microgels synthesized at 5 min of ultrasound time and (**B**) 20% amplitude, and (**C**) 40% amplitude. 20 min of ultrasound time at (**D**) 20% amplitude (polydispersity index (PDI): 0.293 ± 0.031) and (**E**) 40% amplitude (PDI: 0.35 ± 0.071). (**F**) The hydrodynamic diameters of PAA/Schizo microgels synthesized at the same described conditions obtained by dynamic light scattering. Data shown is the mean of three independent measurements ± standard deviation (SD).

The influence of the amplitude percentage and ultrasound time for the formation of spherical particles was next determined. Figure 2A shows a picture of the suspensions formed by varying the mentioned conditions. It can be observed that control polymer (PAA) is characterized by a transparent suspension while hybrid PAA/Schizo microgels synthesized at different amplitudes and ultrasound times became turbid, indicating the reaction of PAA and *Schizochytrium* sp. CWF. 

The morphology and hydrodynamic size of PAA/Schizo microgels were evaluated by TEM and DLS to select the best synthesis conditions. The synthesis performed at 20% and 40% amplitude and 5 min ultrasound time led to the formation of structures without defined morphology (Figure 2B,C). But increasing the ultrasound time (20 min) allowed to obtain spherical particles at either 20 or 40% amplitude (Figure 2D and Figure 2E, respectively). A more homogeneous sized particles were obtained at the lower amplitude (PDI: 0.293 ± 0.031) when compared those obtained at high amplitude (PDI: 0.35 ± 0.071), in which high polydispersity was observed (figures inset). These findings point out sonication time as a crucial factor for the formation of microgels particles while amplitude might help to control particle size dispersion.

The hydrodynamic diameter of PAA/Schizo microgels was evaluated by DLS at different reaction conditions; increasing sonication time and amplitude percentage led to the formation of smaller particles (Figure 2F). Li et al. [39] reported PAA/calcium phosphate hybrid nanogels synthesized by ultrasound, observing a decrease in particle size as long as sonication time and amplitude were increased. In this study, conditions determined as convenient for the synthesis of PAA/Schizo microgels comprised 20 min of ultrasound treatment at a 20% amplitude, which allowed the production of more homogeneous spherical shaped particles with a narrow size distribution; which is a desirable goal to achieve a more consistent biological activity since particle size influences the uptake by immune system cells [27,58]. 

The stability of PAA/Schizo microgels was determined by measuring PDI of the samples prepared at 20 min of ultrasound time and 20% amplitude (particles observed in Figure 2D). The results showed a substantial increase in PDI values as well as in particle size along the time. This effect was attributed to particle breaking and polymer aggregation. The lack of stability of hybrid PAA/Schizo particles in aqueous suspension might indicate that the synthesis conditions selected to prepare the microgels mainly promoted intermolecular interactions between PAA and microalgae CWF, which are easily broken by hydrolysis in aqueous solution due to their intrinsic weak nature. 

**Figure 3:**
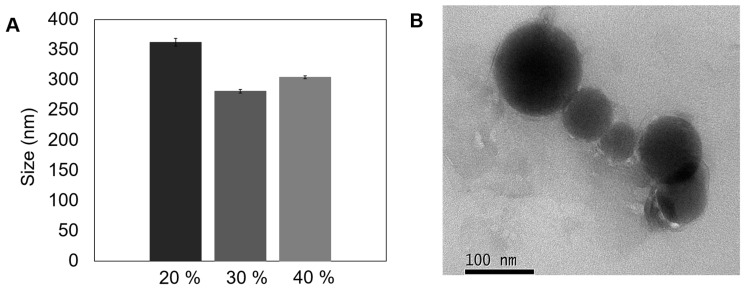
Morphological characterization and particle size of polyacrylic acid/*Schizochytrium* sp. (PAA/Schizo) microgels obtained by inverse emulsion assisted by ultrasound. (**A**) Size of synthesized microgels at different amplitudes. (**B**) Transmission electron microscopy (TEM) image of microgels syntesized at 30% amplitude for 20 min. Data shown is the mean of three independent measurements ± standard deviation (SD).

Therefore, stability and homogeneity of PAA/Schizo microgels were achieved thanks to the formation of amide bonds between PAA and microalgae CWF, in a reaction induced in a water-in-oil (W/O) microemulsion prepared with non-toxic compounds (coconut oil and PGPR as the surfactant). Under this synthesis approach PAA concentration and ultrasound time were maintained constant at 0.3 mg/mL and 20 min, respectively, and the effect of amplitude (20, 30, 40%) on particle size and shape was determined (Figure 3). 

The 30% amplitude treatment (Figure 3A) allowed to obtain microgels with the smallest average hydrodynamic size (281 ± 2.9 nm) and PDI value (0.21 ± 0.013), compared with microgels synthesized at 20% (362 ± nm, PDI 0.35 ± 0.031) and 40% (304 ± 2.4 nm, PDI 0.25 ± 0.01). Ruiz et al. [59] recently analyzed the influence of sonication parameters on particle size and PDI of polylactic acid (PLA) nanoparticles, observing that an increase in sonication power led to a decrease in particle size. The authors argue that the hot spots created by the transitory cavitation causes a reduction in the size of the droplets and the progression of the treatment led to breakdown of smaller drops. However, particle size and PDI increased when a 40% amplitude was applied, an effect that could be explained by the increment in temperature after constant sonication, leading to a decrease in the energy system that causes particle aggregation.

The reproducibility of the microemulsion synthesis method was determined by DLS measuring the particle size in 20 samples from independent reactions performed at 30% amplitude, observing an average diameter of 288 ± 39 nm. Average size here obtained is desirable for biological applications due to particles > 100 nm are uptake by peripheral antigen presenting cells (APCs) and then migrate and maturate to present the antigen to T cells [27,58]. Morphological characterization of this representative PAA/Schizo microgel in dried form is shown in Figure 3B, which revealed a compact structure, with spherical-like shape particle and a diameter below 100 nm. The difference in particle size observed between DLS and TEM is attributed to the dehydration effect of the nanoparticles upon TEM analysis.

The comparison of the two synthesis methods revealed that the inverse microemulsion allows the production of more nanostructured and smaller microgels. Thus the final synthesis conditions for further experiments were set as follows: 0.3 mg/mL PAA, 6 mg CWF, 30% amplitude, and 20 min ultrasound time.

### 3.2. Infrared Spectroscopy Shows Modifications in the Chemical Structure of PAA/Schizo Microgels

**Figure 4:**
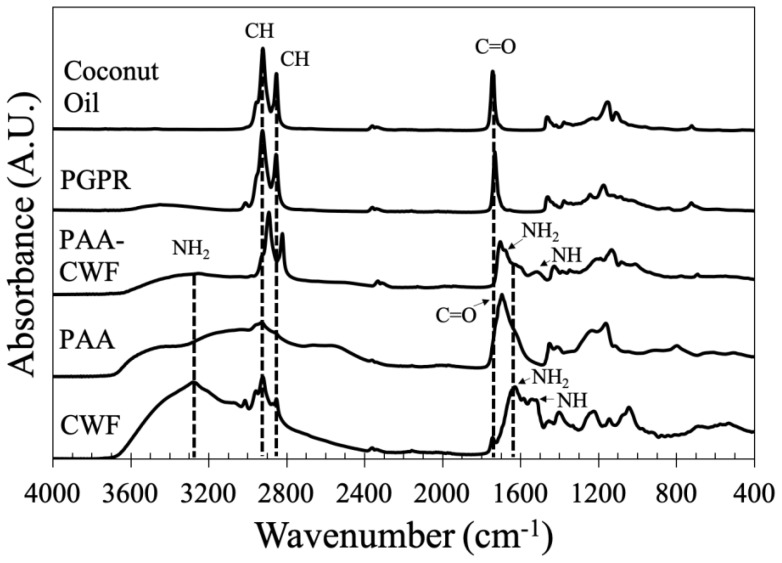
FT−IR spectra of polyacrylic acid/*Schizochytrium* sp. (PAA/Schizo) microgels and their components: cell wall fraction (CWF), polyacrylic acid (PAA), polyglycerol polyricinoleate (PGPR) and coconut oil.

The evolution of hybrid polymer was monitored by FT-IR at the different synthesis steps and compared with the pure components. The infrared spectrum of CWF is shown in Figure 4, where the major absorption bands of the -NH_2_ group are observed (at 3260 and 1620 cm^−1^), while the stretching vibration of –NH is observed at 1515 cm^−1^, both belonging to proteins. In the pure PAA, the strong stretching vibration band of C = O characteristic of the carboxyl group belonging to carboxylic acid is located at 1690 cm^−1^. For the coconut oil and PGPR, the major IR bands characteristic of triglyceride functional groups are observed around 2920 cm^−1^ for C-H asymmetric stretching, 2850 cm^−1^ for C-H symmetric stretching, and 1740 cm^−1^ for C=O stretching. The formation of hybrid PAA/Schizo microgels was confirmed by the IR band centered at 3260 cm^−1^, which belongs to primary amine groups observed in CWF, while the band at 1690 cm^−1^ assigned to the carboxylic acid of PAA became lower, broader, and slightly shifted, indicating modifications in the chemical structure of the polymeric molecule. All these changes suggested the chemical bonding between carboxyl groups of PAA and amine groups of CWF leading to the formation of amide groups, which is further confirmed by the presence of the vibration bands at 1620 and 1515 cm^−1^ corresponding to amide I and II groups, respectively. 

### 3.3. Schizochytrium sp. CWF Improves Thermal Stability of PAA/Schizo Microgels

**Figure 5:**
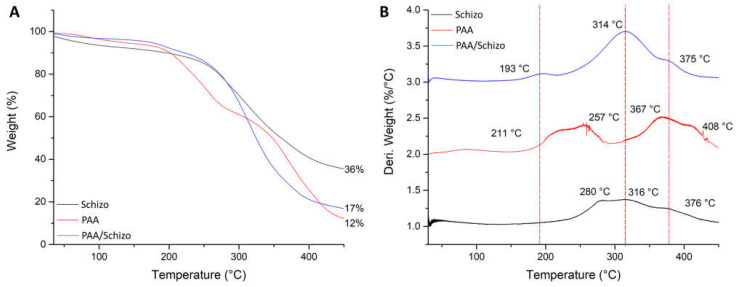
Thermogravimetric analysis (**A**) and derivative thermogravimetric (DTG) curves (**B**) of PAA, *Schizochytrium* sp. biomass and PAA/Schizo microgels. Thermal decomposition of the samples was carried out from 30 to 450 °C with a heating range of 10 °C/min under nitrogen gas flow.

In addition, thermal decomposition of PAA, CWF and PAA/Schizo microgels was evaluated by TGA. As shown in Figure 5A, CFW presented the highest thermal stability with initial weight loss that may correspond to the adsorbed and bound water. A significant weight loss in CWF was observed in the range of 260–400 °C that could be due to the organic molecules such as proteins and carbohydrates present in the biomass [60]. Weight loss in PAA can be observed in two stages: first one ranges between 200–290 °C and can be attributed to the thermal decomposition of carboxyl groups, whereas the second one occurred in the range of 330–450 °C belonging to the breaking of PAA backbone. PAA/Schizo sample showed the initial weight loss at 200 °C corresponding to carboxyl groups degradation followed by a maximum weight loss starting at 250 °C. DTGA thermograms showed differences in the decomposition temperature peaks of the different samples analyzed. PAA/Schizo microgels presented a maximum degradation peak at 314 °C which is not observed in PAA DTGA thermogram. This could indicate the presence of inter- and intramolecular interactions between PAA and CWF. In addition, CFW improved thermal stability at higher temperatures. 

### 3.4. PAA/Schizo Microgels Are Stable at Neutral and Basic pH Values

**Figure 6:**
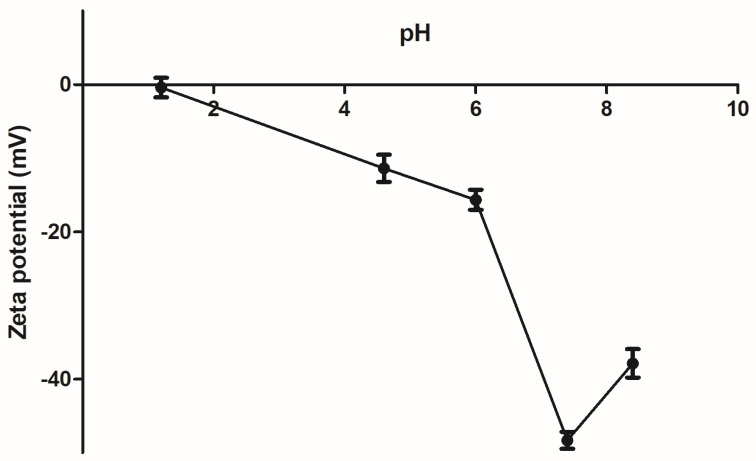
Zeta potential (Ƶ) of polyacrylic acid/*Schizochytrium* sp. (PAA/Schizo) microgels was evaluated at different pH values at 25 °C. Data shown is the mean of three independent measurements ± standard deviation (SD).

On the other hand, stability of the PAA/Schizo microgels in buffered aqueous suspensions was determined in terms of zeta potential (Ƶ). It was found that Ƶ of microgels is pH-dependent (Figure 6). Basic pH confers a negative charge to PAA/Schizo microgels, which is attributed to the deprotonation of carboxylic acid of the polymeric network. The highest negative value (lower than −30 mV) was found between pH 7.4 and 8.4, denoting the high stability of the microgels at physiological pH [61] assuring a good dispersion of the nanoparticles in the suspension.

**Figure 7:**
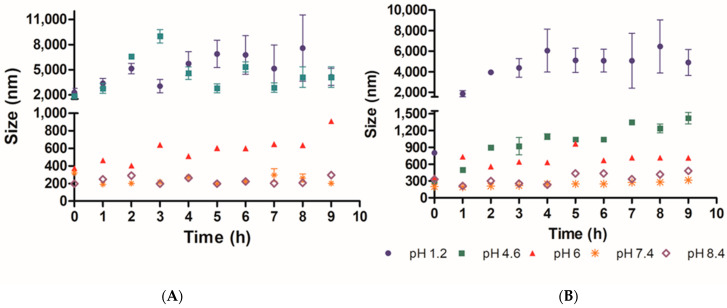
Microgel stability of polyacrylic acid/*Schizochytrium* sp. (PAA/Schizo) microgels was analyzed by determining the changes in particle average size (size) and polydispersity index (PDI) of samples over 9 h, at (**A**) 25 and (**B**) 37 °C at different pH values. PAA/Schizo microgels showed higher stability at 7.4 and 8.4 pH values. Data shown is the mean of three independent measurements ± standard deviation (SD).

In order to determine the suitability of the PAA/Schizo microgels for biomedical applications, their stability was assessed by DLS in buffered aqueous solutions at different pH values at 25 and 37 °C. When incubated at 25 °C PAA/Schizo microgels display a wide size range at pH 1.2 and 4.6 (Figure 7A). PDI also increases over the time at pH 1.2 and 4.6, indicating an increase in nanoparticles size distribution with a final PDI values of 0.68 ± 0.134 and 1, respectively; this behavior is attributed to the lower stability of microgels, which apparently lose their spherical shape and form aggregates at acid pH. PAA/Schizo microgels show good stability with no significant changes in average size and PDI at pH 7.4 and 8.4 at either 25 or 37 °C (Figure 7A,B). When incubating at 25 °C, final PDI values were 0.183 ± 0.026 and 0.239 ± 0.013 for pH 7.4 and 8.4, respectively. These results are in agreement with those obtained in the zeta potential analysis.

PAA have been used to synthesize microgels for drug delivery due to its pH-responsiveness capability [39,62,63]. PAA/Schizo microgels resulted stable at pH 7.4 and 8.4 for a prolonged time at 25 and 37 °C while at acidic pH they dissolved due to the association between the carboxylic groups with the protons of the medium. The hydrogen bonds formed between carboxyl groups and H^+^ at lower pH may lead to aggregates formation after dissolution, which is reflected in an increase in PDI and particle size values; whereas at basic pH values, PAA/Schizo microgels have higher stability and a negative surface charge because of PAA chains deprotonation. These hypotheses are also supported by the non-significative changes observed in particle size and PDI, as well as zeta potential values under −30 mV. Swelling occurs at a pH above the pKa (4.25 for acrylic acid), in this case a 4.6–6 pH range at 37 °C, where microgels are in a swollen state owing to electrostatic repulsion between segments within the particles. When exposed to higher temperatures, average size of PAA/Schizo microgels increase due to the breakage of weaker hydrogen bonds between acrylic acid units [64]. These characteristics make PAA/Schizo microgels a promising material for biomedical applications owing to its compatibility with physiological conditions [65]. 

Morphological and physicochemical characteristics of microgels depend on the synthesis method, cross-linking agents, polymerization degree, among others. In fact, we obtained similar results compared with other studies. Koul et al. [66] reported the synthesis of interpenetrating polymer network (IPN) based on different combinations of gelatin and PAA by inverse miniemulsion, obtaining spherical microgels with a 255 ± 25 nm mean diameter with high stability at basic pH values; Pal et al. [67] also synthesized stable and spherical PAA grafted gelatin microgels of 442 nm in diameter for drug delivery. Chitosan/PAA microgels have also been reported, synthesized by free radical polymerization rendering nanospheres of 114.5 nm in diameter [68]. However, these synthesis methods involve the use of toxic cross-linkers and solvents, which generate hazard residues and imposes the need of a strident purification process of the material to guarantee its safety for biomedical use. Interestingly, our synthesis method avoids the use of toxic reagents and relies in the use of *Schizochytrium* sp. CWF as novel cross-linker, therefore will serve as an attractive vehicle in several biomedical applications. 

### 3.5. PAA/Schizo Microgels Adsorb BSA and Are not Inherently Toxic

**Figure 8:**
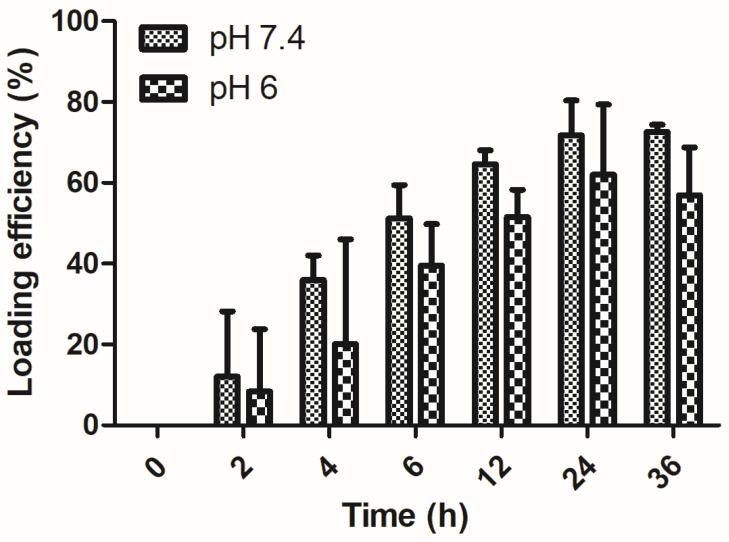
BSA loading efficiency of polyacrylic acid/*Schizochytrium* sp. (PAA/Schizo) microgels. A bovine serum albumin (BSA) solution (2:1) at pH 7.4 and 6. Microgels were incubated at 4 °C with a BSA solution at a 2:1 proportion, and the amount of free BSA in the supernatant was measured at different time points by the Lowry assay using a standard calibration curve. Data shown is the mean of three independent experiments ± standard deviation (SD).

To evaluate PAA/Schizo microgels as antigen delivery vehicles, BSA protein was used as model antigen, which was loaded into microgels by the incubation method. BSA loading efficiency (LE) of PAA/Schizo microgels at different pH are shown in Figure 8. The PAA/Schizo microgels exhibit higher LE (72%) at pH 7.4 compared to pH 6 (57%), which could be due to favored intermolecular bonds at higher pH values. The highest amount for BSA loaded on the microgels occurred after 24 h incubation for both cases; no noticeable increase in loading was observed after this time suggesting saturation of the PAA/Schizo microgels with the antigen. At pH 6 and 7.4, both BSA and PAA/CWF microgels have negative charges so the driving force of loading BSA onto the PAA/Schizo microgels could be through hydrophobic interaction between BSA and the PAA chains [41]. The LC of BSA in the PAA/Schizo microgels at pH 7.4 was 362 µg/mg while at pH 6 was 285 µg/mg. 

The yields obtained in this system are attractive when compared with those reported by Argentiere et al. [69] who synthetized PAA microgels by emulsion polymerization of methyl acrylate and subsequent acidic hydrolysis with a loading capacity of 36 and 8 µg/mg at pH 4.5 and 7, respectively. PAA/Schizo microgels LE and LC also resulted higher than those reported by Morelli et al. [70] who synthetized a hybrid microgel composed by N-vinylcaprolactam and ulvan (algal sulphated heteropolysaccharide) by UV-initiated radical copolymerization; they reported a protein loading of 44 and 22 µg and a LE of 34 and 17%, respectively. PAA/Schizo microgels loading capacity also resulted higher when compared with other nanomaterials such as porous silica nanoparticles (430 nm in size), which exhibited a loading capacity of 41.2 µg BSA/mg nanoparticles [71].

**Figure 9:**
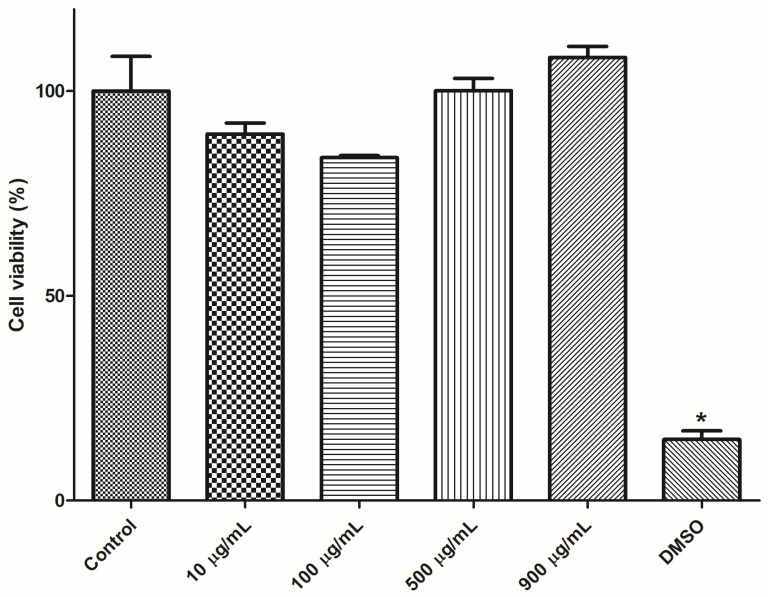
Cytotoxicity assessment for polyacrylic acid/*Schizochytrium* sp. (PAA/Schizo) microgels in mice splenocytes. Cells from BALB/c mice were exposed during 24 h to PAA/Schizo microgels at different concentrations and viability was subsequently measured by the resazurin assay. Vehicle (PBS) and 5% dimethyl sulfoxide (DMSO) were used as controls. Data represent mean ± standard error of the mean (SEM) (n = 3). Statistic differences (* *p* < 0.05) are indicated by the asterisk.

Cell viability of mice splenocytes was investigated by resazurin assay after incubation with PAA/Schizo microgels at different concentration. As shown in Figure 9, cell viability after incubation with the microgels for 24 h resulted higher than 80 % for all tested concentrations (10, 100, 500 and 900 µg/mL) and there was not significative difference when compared with control. Thus, PAA/Schizo microgels could be used as antigen delivery systems due to its good biocompatibility. 

The obtained microgel was safe as it did not exert a significant depletion on cell viability in splenocyte cultures and no obvious toxic effects in immunized mice (weight loss, mortality). This data is critical to continue with the preclinical evaluation of the PAA/Schizo microgels. Other groups have investigated the safety of PAA-based nanomaterials in different cell lines, such as LoVo cells [41,72], L02 human cells [39], and Caco-2 cells [73]. Interestingly, Mahajan et al. [74] reported thermally reversible xyloglucan gels applied for nasal drug delivery, which were obtained from tamarind seed by partial degradation by β-galactosidase. This material induced no damage in the nasal mucosa and achieved a 28.64% increase in the drug bioavailability.

### 3.6. PAA/Schizo Microgels Modified the Immune Response against BSA

**Figure 10:**
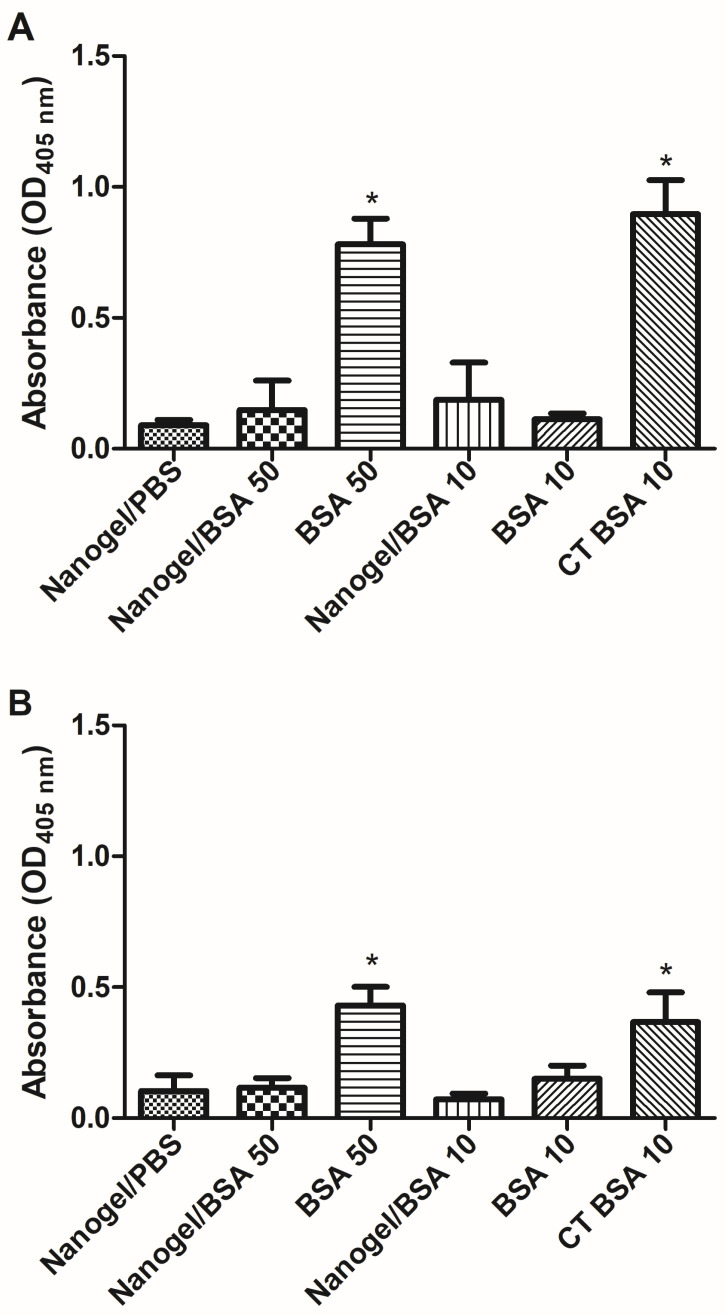
Humoral response analysis induced in test mice upon nasal administration of bovine serum albumin (BSA) soluble or adsorbed in polyacrylic acid/*Schizochytrium* sp. (PAA/Schizo) microgels. (**A**) serum anti-BSA IgG or (**B**) nasal washes anti-BSA IgA levels were measured by ELISA in samples from BALB/c mice immunized with soluble BSA at two different doses: 10 or 50 µg (BSA 50, BSA 10); or embedded in microgels at two doses: 10 or 50 µg (microgel/BSA 50, microgel/BSA 10). One group received 10 µg BSA soluble plus Cholera toxin (CT) as an adjuvant. Mice were subjected to a scheme comprising three intranasal (i.n.) doses. Data represent mean ± standard error of the mean (SEM) (n = 4). Statistical differences (* *p* < 0.05) are indicated by an asterisk (versus the group treated with PAA/Schizo microgels in PBS only).

To investigate the capacity of PAA/Schizo microgels to potentiate the immune response induced against BSA, an initial study was carried out in test mice. An i.n. immunization scheme comprising three weekly doses of BSA (10 or 50 µg of BSA) was implemented. All mice survived during the immunization scheme. IgG serum antibody levels were determined by ELISA, observing that the 50 µg dose of soluble BSA induced significant antibody responses at a similar magnitude to that induced by the 10 µg BSA dose plus CT as adjuvant. Surprisingly, the 50 µg BSA dose included in the PAA/Schizo carrier induced no significant responses (Figure 10A). In terms of the mucosal immune response, IgA levels were determined in nasal washes, observing the same effect: soluble BSA at a 50 µg dose induced significant humoral responses similar to that induced by BSA (10 µg plus CT), whereas BSA (50 µg) absorbed into the PAA/Schizo carrier did not (Figure 10B).

**Figure 11:**
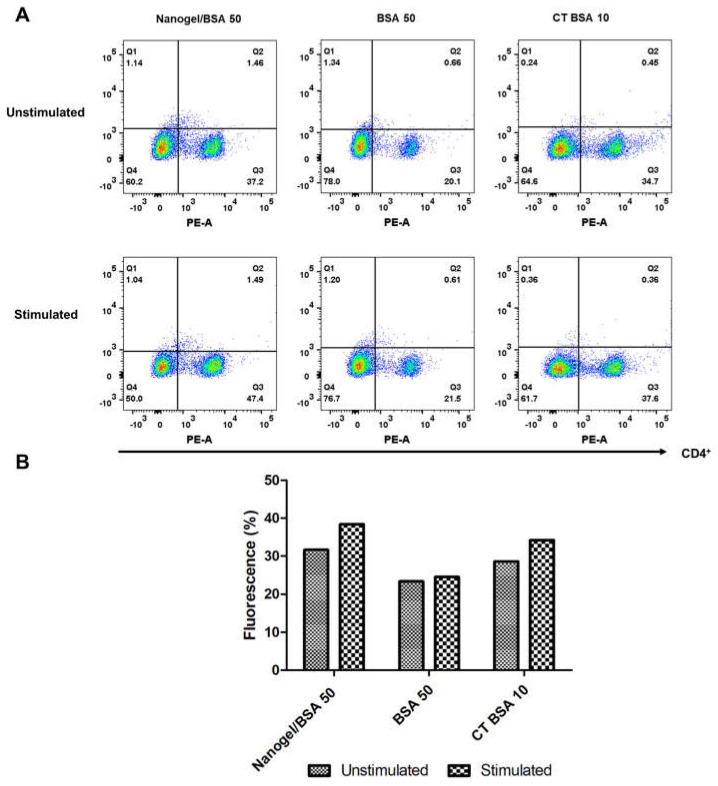
Splenocytes from mice immunized with polyacrylic acid/*Schizochytrium* sp. (PAA/Schizo) microgel + 50 µg bovine serum albumin (Microgel/BSA 50), 50 µg bovine serum albumin soluble (BSA 50) or Cholera toxin + 10 µg bovine serum albumin (CT BSA 10) were incubated for 65 h in either the presence or absences of 2.5 mg/mL BSA soluble and labeled with anti− CD4− PE antibody. (**A**) Lymphocyte-gated regions along with dot plots of representative results are presented. (**B**) Mean percentage of fluorescence is presented. Augment in CD4^+^ T cell proliferation was observed in mice immunized with PAA/Schizo microgel + 50 µg BSA.

To assess whether the CD4^+^ T cells response is differentially induced when the PAA/Schizo microgels are used as antigen carriers, splenocytes were isolated from test mice and subjected to in vitro stimulation with BSA and analyzed by flow cytometry. Results showed that mice immunized with PAA/Schizo microgels + 50 µg BSA have an increased rate of proliferating CD4^+^ T cells upon BSA stimuli compared with unstimulated splenocytes (Figure 11B); whereas proliferating CD4^+^ T cells response of mice treated with 50 µg soluble BSA was significant but lower than that of the mice group immunized with the PAA/Schizo microgel carrying the 50 µg BSA dose. 

By one hand, neither IgG (systemic) nor IgA (nasal mucosa) responses were induced after nasal immunization with 50 or 10 µg BSA doses embedded in PAA/Schizo microgels, whereas immunization with the soluble antigen resulted in significant humoral responses in both compartments. On the other hand, CD4^+^ T−cells proliferation assays using splenocytes revealed significant responses induced by the PAA/Schizo microgel carrying BSA. This differential behavior observed for the humoral and lymphoproliferative responses leads us to speculate on some of the immune mechanisms behind. It is known that upon immunization APCs, like dendritic cells (DCs), present epitopes of antigens on MHC class II (MHC−II) to naïve CD4^+^ T−cells, leading to their activation. In draining lymph nodes, CD4^+^ T−cells can differentiate into effector cells such as Th1 and Th2 or into regulatory T-cells (Tregs) [75]. Tregs play an essential role in development of self−tolerance and they downregulate proinflammatory responses of effector Th cells, and also downregulate the induction of humoral responses [76,77]. 

It has been documented that nasal or mucosal tolerance can be induced by repeated administration of low antigen doses [78]. The slow antigen release presented by PAA-based microgels [41,79] could provide a low antigen uptake by APCs directing CD4^+^ T−cells differentiation into Tregs. PLGA (poly-lactic-co-glycolic acid) nanoparticles are polymeric materials that also show slow release and they have been involved in the induction of tolerance via intranasal. Slütter et al. [80] synthetized N-trimethyl chitosan (TMC) and PLGA-based nanoparticles loaded with OVA; Balb/c mice were nasally immunized with 20 µg OVA loaded in PLGA-based nanoparticles. PLGA and PLGA/TMC nanoparticles showed no significant burst release and up to 80% release of initial OVA in 25 days. Although PLGA (320 ± 17.9 nm, zeta potential −48.2 mV) and PLGA/TMC (448 ± 55.9 nm, zeta potential 24.5 mV) nanoparticles failed to elicit specific antibody response after nasal vaccination. A subsequent study revealed that PLGA nanoparticles (371 ± 17.9 nm, zeta potential −16.8 mV) induced mucosal tolerance after nasal vaccination, which was associated to the CD4^+^ T cell differentiation into FoxP3+ T-cells [81]. A critical perspective for this study is the characterization of the Treg response promoted by the PAA/Schizo microgels.

The functional difference in DCs (immunogenic, tolerogenic) depends on the maturation state and maturation environment. Maturation of DCs is a complex process in where antigen processing and presentation, migration and T-cell co-stimulation are involved [82]. Mature DCs are capable of induce clonal expansion of antigen-specific naïve T cells and their concomitant differentiation into effector T cells. The homeostatic balance of high levels of MCH class II in DCs confers tolerogenic properties rather than immunogenic properties. In addition, anti-inflammatory cytokines such as IL-10 drives differentiation of Tregs, which promotes the production of IL-10 and transforming growth factor (TGF)-β inhibitors of immune response. PLGA particles have been reported not to increase DCs maturation while they prolong the expression of MHC class II on the cell surface of DCs [80,83].

Induction of mucosal tolerance is an important therapeutic approach in autoimmune diseases, allergy and chronic inflammatory disorders [84,85]. Polymeric-based nanomaterials such as PLGA have been used as tolerogenic vaccine carriers in experimental models of autoimmune diseases [86]. Interestingly, Kim et al. [87] administered a single oral dose of PLGA nanoparticles encapsulating type II collagen (CII) (a potential Rheumatoid Arthritis-associated autoantigen) in DBA/1 mice. CII was retained up to 14 days in Peyer’s Patches dome area, where tolerance takes places, moreover, mice were protected from collagen-induced-arthritis. In this context, the PAA/Schizo microgels obtained in the present study are promising candidates for the development of immunotherapy prototypes targeting relevant human diseases whose correlates of protection are related to the downregulation of inflammatory responses. At this point we can only speculate that the enhanced Th response observed in the PAA/Schizo microgels-treated mice and absence of humoral responses could be related to the expansion of Tregs that down regulate the humoral response against BSA. Further research will comprise mechanistic studies that will allow to assess this hypothesis and assessing if this effect is also observed for antigens related to relevant pathologies, such as type I diabetes and atherosclerosis. 

The potential of microgels for vaccination is clearly illustrated by the case of pullulan-based microgels that are under evaluation in clinical trials for the delivery of vaccines against cancer. NY-ESO-1 and HER2 cancer antigens have been associated to complexes of cholesteryl pullulan (CHP) microgels and have induced specific humoral and cellular immune responses [88,89,90]. 

## 4. Conclusions

This study provides a new, sustainable biomaterial based on PAA and *Schizochytrium* sp. CWF as a cross-linker agent, called PAA/Schizo microgels, which is synthesized by an innovative methodology which avoid the use of toxic solvents and crosslinkers. The obtained PAA/Schizo microgels have several attributes, such as spherical shape, high stability at basic pH, no inherent toxicity in mouse splenocytes at concentrations up to 900 µg/mL. The staibilty of the PAA/Schizo microgels is likely due to the strong interactions of CWF and the PAA chains. Furthermore, PAA/Schizo microgels have a high antigen loading efficiency and were deemed functional intranasal antigen delivery vehicle since these induced a differential immune response when compared to the response induced by the soluble antigen. Therefore, PAA/Schizo microgels offer a new alternative to obtain a high value, sustainable antigen delivery system by using a subproduct form algae bioprocessing, with a possible application in tolerogenic immunotherapies. 

## Data Availability

Not applicable.
